# Nonstationary flood coincidence risk analysis using time-varying copula functions

**DOI:** 10.1038/s41598-020-60264-3

**Published:** 2020-02-25

**Authors:** Ying Feng, Peng Shi, Simin Qu, Shiyu Mou, Chen Chen, Fengcheng Dong

**Affiliations:** 1 State Key Laboratory of Hydrology-Water Resources and Hydraulic Engineering, Hohai University, Nanjing, 210098 China; 2 College of Hydrology and Water Resources, Hohai University, Nanjing, 210098 China

**Keywords:** Climate-change impacts, Climate-change impacts, Hydrology, Hydrology, Hydrology

## Abstract

The coincidence of flood flows in a mainstream and its tributaries may lead to catastrophic floods. In this paper, we investigated the flood coincidence risk under nonstationary conditions arising from climate changes. The coincidence probabilities considering flood occurrence dates and flood magnitudes were calculated using nonstationary multivariate models and compared with those from stationary models. In addition, the “most likely” design based on copula theory was used to provide the most likely flood coincidence scenarios. The Huai River and Hong River were selected as case studies. The results show that the highest probabilities of flood coincidence occur in mid-July. The marginal distributions for the flood magnitudes of the two rivers are nonstationary, and time-varying copulas provide a better fit than stationary copulas for the dependence structure of the flood magnitudes. Considering the annual coincidence probabilities for given flood magnitudes and the “most likely” design, the stationary model may underestimate the risk of flood coincidence in wet years or overestimate this risk in dry years. Therefore, it is necessary to use nonstationary models in climate change scenarios.

## Introduction

The term flood coincidence is used to denote the simultaneous occurrence of floods in two (or more) rivers. The coincidence of flood flows in a mainstream and its tributaries may lead to catastrophic floods^1^. Therefore, assessing the risk of flood coincidence for the main river and its tributaries is critical for flood control and water project operations. As flood events are characterized by flood occurrence dates and flood magnitudes, both of these factors should be taken into account when analyzing the flood coincidence risk^2^. In addition, the analysis of flood coincidence involves at least two rivers. For these reasons, a multivariate hydrological analysis is needed that considers the dependence among flood variables^3–5^.

Traditionally, multivariate probability distributions are derived using various assumptions, e.g., the same type of marginal distribution or independence of the variables is assumed^6^. In addition, considering multivariate models from this traditional perspective, mathematical formulations are often complicated when more than two variables are involved^7^. For these reasons, a new method of determining the multivariate probability distribution based on copula functions was proposed by Sklar^8^. The copulas describe and model the dependence structure among random variables, independently of the margins involved. Due to their flexibility of construction, copula functions have been widely used in multivariate hydrological frequency analyses in recent years^9–17^, especially in flood coincidence risk analyses^1,18,19^.

The risk of flood coincidence has mainly been analyzed under the stationarity assumption. In other words, both the marginal distribution and the copula function are modeled with fixed moments and parameters^18–20^. However, climate change and anthropogenic activities have changed the statistical characteristics of hydrological series and the dependence structure of the variables^21,22^. As a result, increasing attention is being paid to the development of nonstationary multivariate models with copula functions^23–25^. In the univariate case, nonstationary models have been widely applied^26–28^. Liang, *et al*.^29^ grouped nonstationary flood frequency methods into two types: indirect and direct methods. Direct methods have been widely used because they do not require the restoration of hydrological series. In the multivariate case, copulas have been used to describe the dependence structure of different series^30–32^. Chebana, *et al*.^33^ discussed the use of copula functions with time-varying parameters in the case of a changing dependence structure for the investigated variables. Sarhadi, *et al*.^34^ defined the copula parameter as a deterministic function of time. Jiang, *et al*.^32^ compared time-varying copula models with time or a reservoir index as the covariate. However, to the best of our knowledge, few studies in the hydrological field have defined the form of the copula parameter as an autoregressive moving average model (ARMA), which can capture variation in dependence^35^.

**Figure 1:**
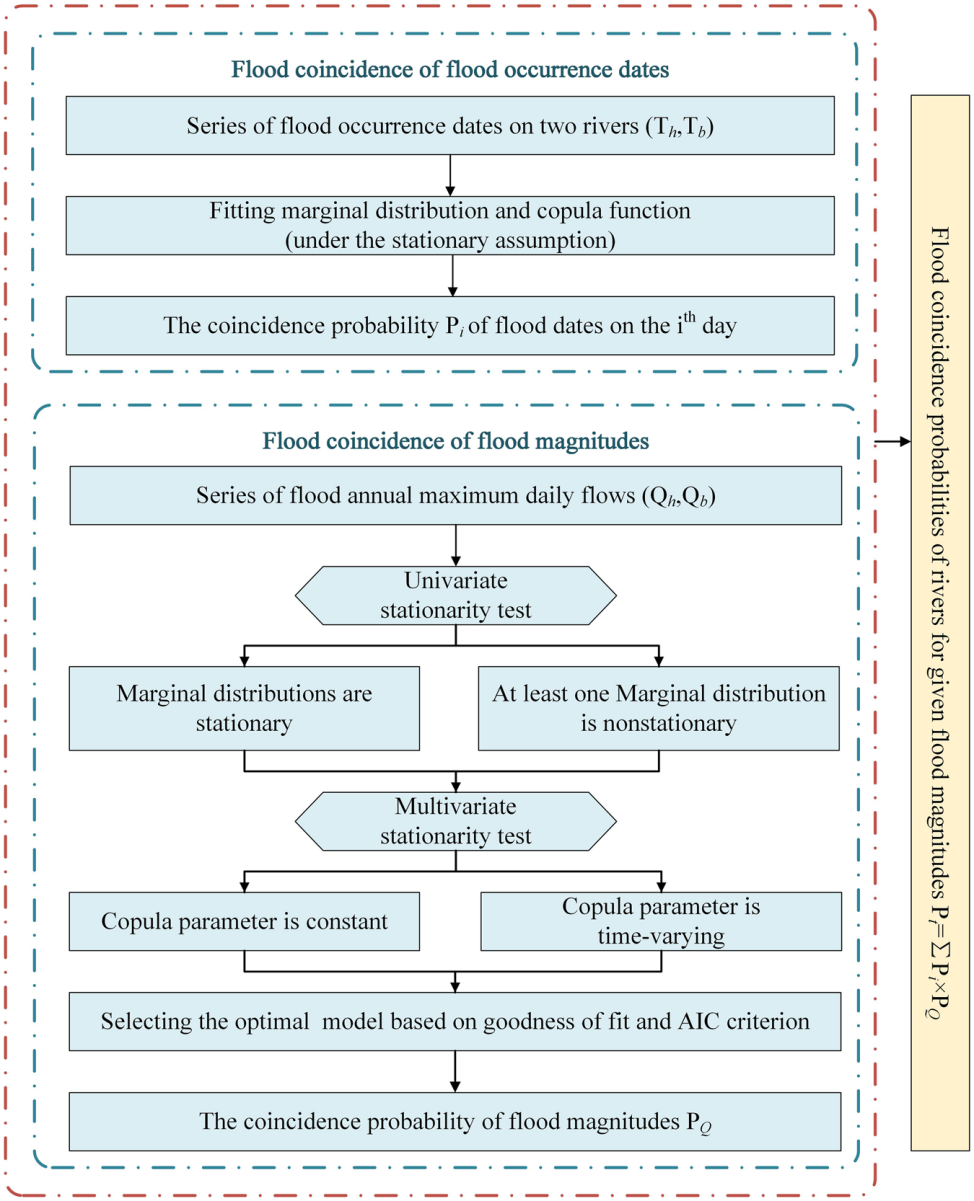
The procedure used to develop the flood coincidence model.

The objective of this study is to apply a time-varying copula to analyze the flood coincidence risk of rivers, considering the nonstationarity of flood magnitudes and the dependence structure among variables (Fig. 1). For this purpose, the mixed von Mises distribution was used as the marginal distribution of flood occurrence dates. Then, the Generalized Additive Models for Location, Scale, and Shape (GAMLSS) model^36,37^ (with rainfall as the covariate in this study) was selected to obtain the marginal distribution of flood magnitudes. Finally, a static copula and a time-varying copula were chosen as candidates to obtain the joint distribution of flood occurrence dates and flood magnitudes. In this study, we (1) assess the nonstationarity of the flood magnitudes and the dependence structure among variables; (2) fit the marginal distributions of flood occurrence dates and flood magnitudes; (3) develop a joint distribution of flood occurrence dates and flood magnitudes; and (4) discuss the risk of flood coincidence by considering annual coincidence probabilities for given flood magnitudes and the “most likely” design^38^.

## Study Area and Data

**Figure 2:**
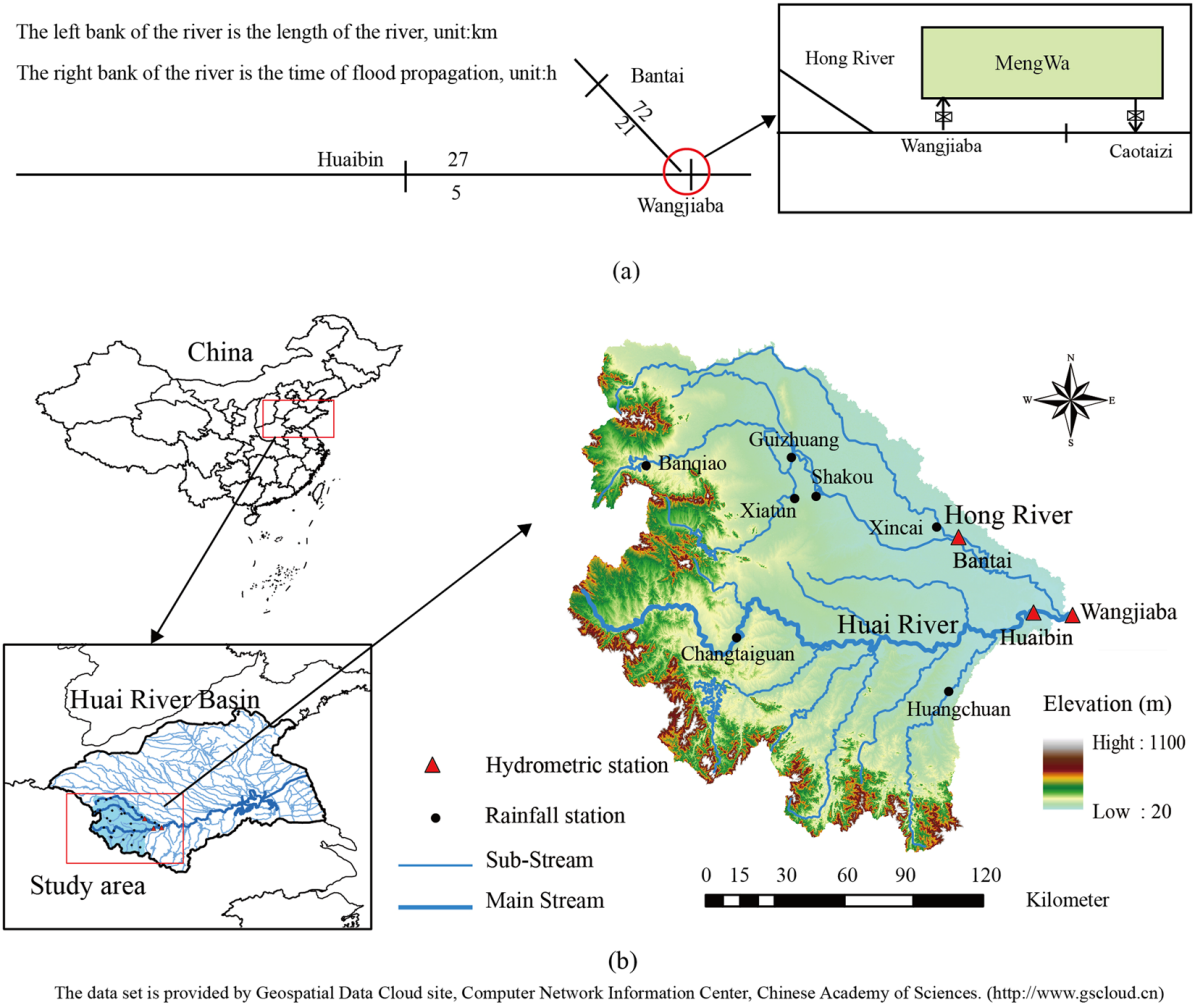
Map of the study area and gauging stations.

The Huai River Basin (N30°55′–36°36′, E111°55′–121°25′), which is composed of many tributaries, is the sixth largest river basin in China. The basin is located in the transitional zone between semiarid and semihumid climates (Fig. 2). Previous studies have shown that extreme rainfall has increased at most stations in the Huai River Basin^39–41^, resulting in more turbulent flows in the main stream and its tributaries during the flood season. Therefore, it is necessary to analyze the risk of flood coincidence in the Huai River Basin.

The study area refers to the upper reaches of the Huai River Basin above the Wangjiaba station, with an area of 2.82 × 10^4^ km^2^ (Fig. 2). The proposed method was applied for flood coincidence analysis for two rivers within the region: one is the upper reach of the main stream of the Huai River, with a catchment area of 1.58 × 10^4^ km^2^, and the other is the Hong River, which has a catchment area of 1.15 × 10^4^ km^2^. The length of the channel from Huaibin to Wangjiaba is 27 km, which drains an area of 900 km^2^. There are seven rainfall stations in the study area: Banqiao (BQ), Guizhuang (GZ), Xiatun (XT), Shakou (SK), Xincai (XC), Changtaiguan (CTG) and Huangchuan (HC). The reason for choosing these two rivers is that coincident flooding on the Huai River and Hong River may generate flood peaks, which can threaten the flood control at Wangjiaba station. The flood control of the Wangjiaba section mainly depends on the Mengwa flood detention basin. When a flood occurs, the diversion gate of the Wangjiaba sluice is opened to discharge the flood into Mengwa, and the outflow of Mengwa is controlled by the Caotaizi sluice (Fig. 2a).

In this study, the annual maximum daily flow (AMDF) of each river (${Q}_{h}$ for Huai River and ${Q}_{b}$ for Hong River) and the occurrence dates (${T}_{h}$ for Huai River and ${T}_{b}$ for Hong River) were obtained using the annual maximum method (AM method). Rainfall data were derived from daily data collected at seven rainfall stations in the study area from 1959 to 2015 (Table 1). Then, the average rainfall in the catchment areas of the two rivers was obtained using Thiessen polygons^42,43^, and the annual maximum rainfalls ${P}_{h}$ and ${P}_{b}$ were sampled from the average rainfall by the AM method and used as covariates. All data were obtained from hydrological yearbook.

**Table 1:** Information on the two rivers in the Huai River Basin.

River	Catchment Area(km^2^)	Hydrometric station		Rainfall station	
		Name	Record of length	Name	Record of length
Hong River	11500	Bantai	1959–2015	BQ,XT,GZ,SK,XC	1959–2015
Huai River	15800	Huaibin	1959–2015	CTG,HC	1959–2015

The flood propagation time of each section in the study area was obtained from the Huai River Water Resources Commission (Fig. 2a).

## Result and Discussion

Before selection of the model for frequency analysis, nonstationarity evaluations (including of the flood magnitude series and dependence structure among series) should be performed. The nonstationarity can be assessed by change point analysis^44–46^ and trend analysis. In this study, the change point was detected by the distribution-free cumulative summation test (CUSUM)^47^. The trend analysis was performed based on the nonparametric Mann-Kendall (MK) test^33^.

**Figure 3:**
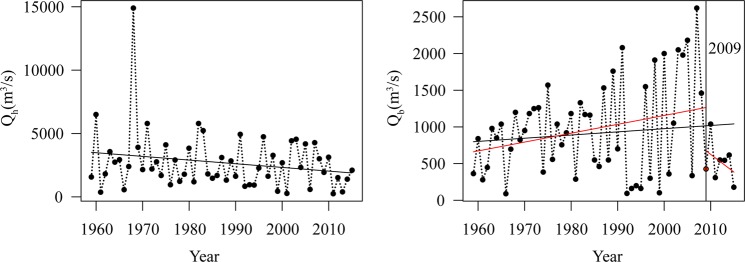
Time series of ${{\boldsymbol{Q}}}_{{\boldsymbol{h}}}$ and ${{\boldsymbol{Q}}}_{{\boldsymbol{b}}}$ data. The vertical solid line indicates the possible mean change point, and the solid red lines indicate the trends before and after the change point.

The results of the change point test show that the mean of ${Q}_{h}$ is constant, and the variance of ${Q}_{h}$ displays an abrupt change in 2009. For ${Q}_{b}$, the change point in the mean occur in 2009, and the change point in the variance is observed in 1985. In addition, a change point in the dependence structure between ${Q}_{h}$ and ${Q}_{b}$ occur in 2010. According to the MK test, the series of ${Q}_{h}$ presents a significant downward trend at the 0.05 significance level. For ${Q}_{h}$, there is a significant upward trend (Fig. 3).

The above analyses demonstrate that the flood magnitudes in the two rivers and the dependence structure are nonstationary. In additions, the flood occurrence dates in the two rivers and the dependence structure between them are both stationary.

### Estimation of marginal distribution

#### Marginal distribution of flood occurrence dates

In this study, the mixed von Mises distribution with constant parameters was selected as the marginal distribution of the flood occurrence dates. The parameters of the mixed von Mises distribution were estimated by the maximum likelihood method. Table 2 summarizes the values of parameters and the goodness-of-fit results for the mixed von Mises distribution of flood occurrence dates for the two rivers: The p-values of the KS test were both larger than 5% (Table 2), which supports the validity of the assumed models.

**Table 2:** Parameters and goodness-of-fit results for the mixed von Mises distribution.

Gauging station	Parameters									*p*-Value
	${\mu }_{1}$	${\mu }_{2}$	${\mu }_{3}$	${k}_{1}$	${k}_{2}$	${k}_{3}$	${p}_{1}$	${p}_{2}$	${p}_{3}$	
Huaibin	3.01	5.06	/	1.33	3.22	/	0.93	0.07	/	0.75
Bantai	0.34	3.03	4.36	1.13	4.17	2.42	0.28	0.42	0.30	0.81

The *p*-value is the approximate Monte Carlo goodness-of-fit test *p*-value (based on Kolmogorov–Smirnov statistics).

**Figure 4:**
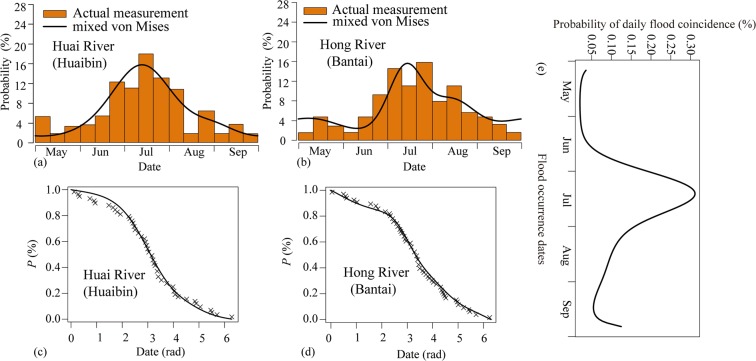
Fitting plots of the mixed von Misses function (**a**–**d**) and the coincidence probabilities for flood occurence dates (**e**).

The frequency histograms of flood occurrence dates fitted by the mixed von Mises distribution are presented in Fig. 4(a,b). The marginal cumulative distribution function (CDF) curves of the flood occurrence dates are shown in Fig. 4(c,d), in which the lines (theoretical distribution) intersect with the observed empirical frequencies. Figure 4(a–d) indicate that the theoretical distribution fits the observed data well. In additions, the highest relative frequencies for the two rivers both occur in July, indicating the floods in these two rivers are more likely to occur during this period.

#### Marginal distribution of flood magnitudes

In this section, both stationary models and nonstationary models with a rainfall covariate were applied to build the marginal distributions of flood magnitudes in the two rivers. Five probability distributions, including the gamma, Weibull, lognormal, Gumbel, and general extreme value (GEV) distributions, were selected as the candidate marginal distributions (Table S1).

Under the stationary assumption, the results of the five distributions in fitting the two series are presented in Table S2. According to the Akaike information criterion (AIC) minimization method, the gamma distribution with constant parameters is the optimal distribution for $\,{Q}_{h}$, and the Weibull distribution with constant parameters is the best distribution for ${Q}_{b}$.

Under the nonstationary assumption, a series of statistical analyses (Table S3) indicate that the Weibull distribution with a rainfall covariate is the best-fitted distribution for ${Q}_{h}$ and that the gamma distribution with a rainfall covariate is the best choice for ${Q}_{b}$.

**Figure 5:**
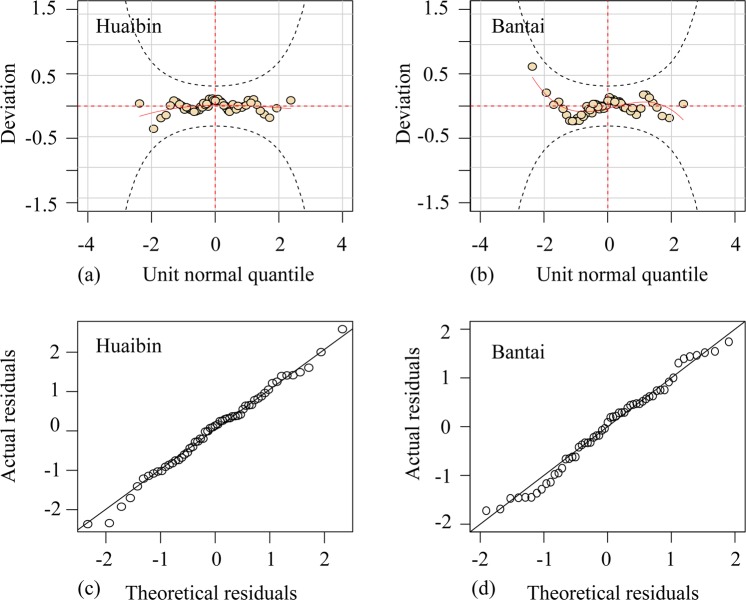
Fitting plots of residual detection at two stations with the GAMLSS model under the nonstationary assumption (**a**–**b**) and linear regression normal QQ diagram (**c**–**d**).

Table 3 summarizes the performance of the four optimal distributions in fitting the two series. According to AIC minimization method, nonstationary models provide a better fit than stationary models for both ${Q}_{h}$ and ${Q}_{b}$. Consequently, the nonstationary models with a rainfall covariate are selected as the marginal distributions of flood magnitudes. The worm plots for the selected models [Fig. 5(a,b)] show that all the points fall in the 95% confidence interval (i.e., the upper and lower gray dotted lines). In the quantile-quantile (QQ) plots [Fig. 5(c,d)], all the points are basically distributed along a straight line at a 45 degree angle. Figure 5 indicates that the actual residuals of the selected models are in good agreement with the theoretical residuals.

**Table 3:** Performance of the four optimal distributions in fitting the two series.

Series	Type	Distribution	Distribution parameters		*p*-Value	AIC
			$\mu$	$\theta$		
*Q*_*h*_	Stationary	GA	1856	1.455	0.73	1010
	Nonstationary	WEI	*exp*(7.894)	*exp*(0.607 − 0.065*P*_*h*_)	0.89	946
*Q*_*b*_	Stationary	WEI	1018	1.486	0.57	884
	Nonstationary	GA	*exp*(5.440 + 0.308*P*_*b*_)	*exp*(−0.024 − 0.154*P*_*b*_)	0.32	845

The *p*-value is the approximate Monte Carlo goodness-of-fit test *p*-value (based on Kolmogorov–Smirnov statistics).

#### Estimating the copula parameter

Before the selection of copula functions, the dependence structures among the flood magnitudes and occurrence dates for the two rivers were explored. The Kendall coefficient and Spearman coefficient were used to perform the independence test^48^, as shown in Table 4. The results indicate that ${Q}_{h}$ and ${Q}_{b}$ are significantly correlated at the 5% significance level. However, the coefficient between the flood magnitude and occurrence date in the same river is close to zero, which suggests that these two variables are independent.

**Table 4:** The pairwise correlation coefficients of flood magnitudes and flood occurrence dates.

Coefficients	(*T*_*h*_, *T*_*b*_)	(*Q*_*h*_, *Q*_*b*_)	(*T*_*h*_, *Q*_*h*_)	(*T*_*b*,_ *Q*_*b*_)
Pearson	**0.66**	**0.43**	0.0046	0.0490
Kendall	**0.48**	**0.56**	−0.0123	0.0437
Spearman	**0.62**	**0.78**	−0.0191	0.0605

Three candidate copulas with constant parameters were used for modeling the dependence structure of flood occurrence dates (${T}_{h}$ and ${T}_{b}$), including the Gumbel, Frank, and Clayton copulas. Table 5 summarizes the values of parameters and the goodness-of-fit results for these copulas. According to the goodness-of-fit test results (based on Cramér–von Mises statistics), the Clayton copula is not adequate for modeling the bivariate features of the flood occurrence dates (*p*-value is less than 0.05). In terms of the minimum AIC, the Gumbel copula with ${\theta }_{c}=2.077$ is the best choice.

**Table 5:** Parameters and goodness-of-fit test for candidate copulas in modeling the dependence structure of flood occurrence dates.

Copula	Parameters(s.e.)	AIC	*p*-Value
Gumbel	2.077(0.267)	−37.51	0.382
Clayton	1.421(0.357)	−24.47	0.004
Frank	5.498(0.944)	−28.09	0.141

The values in parentheses indicate estimated standard errors; the approximate *p*-values (via a multiplier method) of the Cramér–von Mises goodness-of-fit test for copulas are also shown.

**Figure 6:**
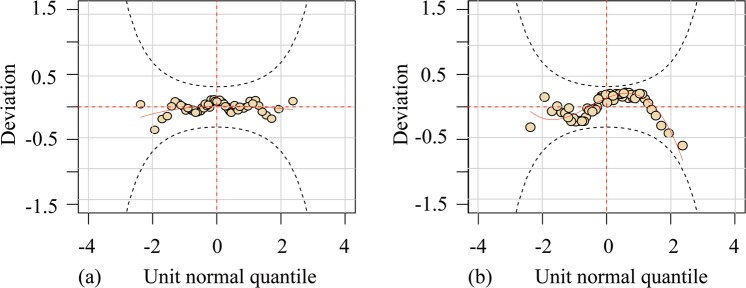
Worm plots of the goodness-of-fit for the time-varying Frank copula: (**a**) worm plot of Rosenblatt’s probabilities of integral transformation for Z_1_; (**b**) worm plot of Rosenblatt’s probabilities of integral transformation for Z_2_.

Considering the nonstationarity of the dependence of flood magnitudes, the time-varying copulas were used as candidates as Eq. (1). The parameters of time-varying copulas were described and estimated as Eqs. (8–9). Table 6 summarizes the values of parameters and the goodness-of-fit results for the optimal copulas under stationary and nonstationary assumptions for modeling the dependence structure of the flood magnitudes (${Q}_{h}$ and ${Q}_{b}$). As Table 6 shows, the P-KS values of Z_1_ and Z_2_ and the P-Kendall values are larger than 0.05, which supports the validity of the assumed models from a statistical perspective. The time-varying Frank copula was selected to model the dependence structure of flood magnitudes by comparing the AIC values. Figure 6 shows the worm plots for the goodness-of-fit test of the Frank copula with time-varying parameters; notably, all points fall within the 95% confidence interval, indicating satisfactory fitting performance for the selected copula model.

**Table 6:** Parameters and goodness-of-fit results for the Frank copulas in modeling the dependence structure.

Type	Copula	Parameter form	Parameters	AIC	*p-*KS of Z_1_	*p-*KS of Z_2_	*p*-Kendall
Stationary	Frank	${\theta }_{c}$	7.039	−42.86	0.73	0.54	0.319
Nonstationary	Frank	$[\omega ,\beta ,\alpha ]$	[1.68, −3.25, 2.90]	−43.98	0.89	0.48	0.332

The *p*-KS (Z_1_) and *p*-KS (Z_2_) are *p*-values of the KS test for the two Rosenblatt’s probabilities integral transformations Z_1_ and Z_2_, which should be uniformly and independently distributed on [0, 1]. The *p*-Kendall is the *p*-value of the Kendall rank correction test for Z_1_ and Z_2_.

#### The risk of flood coincidence

On the basis of marginal distributions and copula functions, the risk of flood coincidence was analyzed from the three following three perspectives.

#### Coincidence probabilities for flood occurrence dates

With the mixed von Mises distribution and copula function, the joint distribution of flood occurrence dates was determined in order to calculate the daily probability of flood coincidence for flood occurrence dates, as described in Eq. (10). The annual coincidence probability of the flood occurrence dates ${P}_{T}=0.152$ was obtained by Eq. (11). According to the observed data, nine floods occurred simultaneously in the two rivers during the 57-year study period. The statistical coincidence probability of the measured data is 0.175, which is close to the model calculation result.

The daily probability of flood coincidence for flood occurrence dates was plotted as shown in Fig. 4(e). The highest probabilities of flood coincidence occur in mid-July, which suggests a high combination risk. Hence, the Wangjiaba flood diversion sluice needs to discharge the floods to Mengwa for flood control during this period. The coincidence probabilities are close to zero from May to mid-June, which indicates that the flood coincidence is extremely low in the two rivers during this period. Therefore, it is possible to open the Caotaizi escape sluices to discharge the flow in the Mengwa flood detention basin during this period.

#### Annual coincidence probabilities for given flood magnitudes

In this section, the annual coincidence probability for given flood magnitudes is calculated. According to the investigation of the pairwise dependence structures, the occurrence dates and magnitudes of floods are independent of each other. Hence, we employ Eq. (12) to describe the joint distribution of the flood magnitudes. Moreover, the annual flood coincidence probability for given flood magnitudes was calculated by Eq. (13).

**Figure 7:**
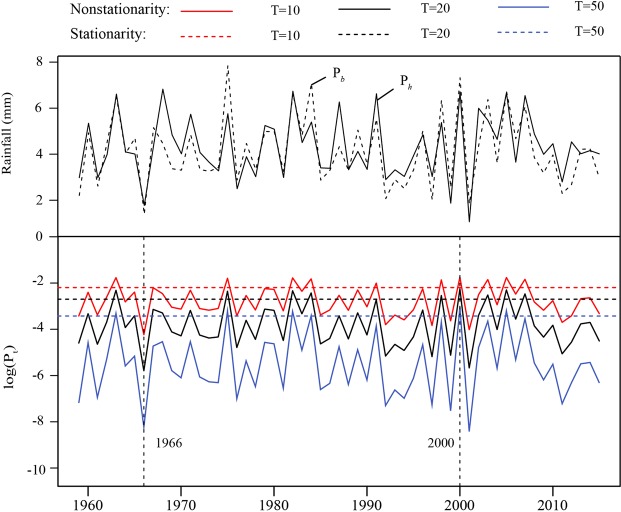
The coincidence probabilities of the Huai River and Hong River and the corresponding rainfall data.

To compare the difference in flood coincidence under stationary and nonstationary conditions, the coincidence probability ${P}_{T}$ in Eq. (13) was calculated under the stationary and nonstationary hypotheses. The values of ${q}_{h}$ and ${q}_{b}$ of the two rivers were the design flows for the $t$-year univariate return period under the stationary assumption. Here, we used $T=(10,20,50)$ years as examples, and the annual coincidence probability for a given flood magnitude and the corresponding variations are presented in Fig. 7. For ease of visualization, the vertical coordinate was set to logarithm of the coincidence probabilities.

Our results indicate that the coincidence probability is constant under the stationary condition. However, under the nonstationary condition, the coincidence probabilities for the flood magnitude fluctuate each year over a wide range. The coincidence probabilities for the flood magnitude display variational processes that are consistent with those of rainfall, reflecting the positive correlation between these factors (Fig. 7). In addition, a series of statistical analyses shows that the nonstationary multivariate model performs better than the stationary multivariate model. The probabilities under the stationary assumption may underestimate the risk of flood coincidence in wet years and overestimate this risk in dry years.

The coincidence probabilities in the case of $T=50$ year are close to zero under both the stationary and nonstationary assumptions, which indicates that the coincident flooding is less likely to occur for large flood events than for other events.

#### Flood coincidence risk in the “most likely” design

**Figure 8:**
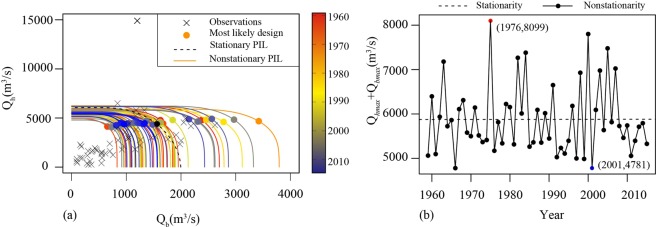
A comparison of the most likely scenario for a specific conincidence probability under the stationary condition and nonstationary condition: (**a**) the plot of PILs and “most likely” design; (**b**) the plot of combined flows of *Q*_*hmax*_ and *Q*_*bmax*_.

The “most likely” design in this section provides the most likely scenario for a specific coincidence probability. According to Eq. (13), the joint exceedance probability-isolines (PILs) for each year can be drawn assuming that the coincidence probability ${P}_{t}$ is a definite value. Here, taking ${P}_{t}=0.01$ as an example, the PILs derived from the stationary model and nonstationary model are shown in Fig. 8(a). The “most likely” design for ${Q}_{hmax}$ and ${Q}_{bmax}\,\,$in each year considering the definite probability can also be obtained according to Eqs. (14–15), and the corresponding results are denoted by color points in Fig. 8(a).

Under nonstationary conditions, the isoline is constantly oscillating over time while it is a fixed curve under stationary conditions. The “most likely” design of ${Q}_{hmax}$ ranges from 4050 m^3^/s to 5035 m^3^/s and that of ${Q}_{bmax}$ ranges from 738 m^3^/s to 3625 m^3^/s. This difference indicates that ${Q}_{b}$ is more susceptible to environmental change than ${Q}_{h}$. The combined flows of ${Q}_{hmax}$ and ${Q}_{bmax}$ in different years are shown in Fig. 8(b) and rang from 4781 m^3^/s to 8099 m^3^/s, which may lead to flood peaks downstream. Hence, the combined flows calculated based on the “most likely” design have a certain reference significance for flood predictions downstream in Wangjiaba. Therefore, it is necessary to consider the flood coincidence between the Hong River and Huai River and take necessary measures to alleviate the pressure on downstream flood control projects.

## Conclusions

Flood coincidence analysis plays an important role in flood risk analysis. This study proposed a nonstationary multivariate model to solve the flood coincidence problem in a changing environment. The proposed model was developed using the mixed von Mises distribution, the GAMLSS model and a copula function. The main conclusions are presented as follows.

First, the probabilities of flood coincidence on two rivers show that coincident flooding is more likely to occur in mid-July than in other periods (Fig. 4e). Hence, it is possible to discharge these floods to Mengwa in this period to alleviate the downstream flood control pressure.

Second, both the flood magnitudes and their dependent structure are verified to be nonstationary, and the nonstationary multivariate model of flood magnitude performs better than the stationary model. The coincidence probability for large floods (i.e., under the situation of $\,T=50$ year) is nearly equal to zero, which indicates that coincident flood events are more likely to occur for medium-scale or small-scale floods.

Finally, the combined flow under stationary and nonstationary conditions can be obtained from the corresponding “most likely” design (${Q}_{hmax}$ and ${Q}_{bmax}$), which provides a basis for downstream flood safety. The range of ${Q}_{bmax}$ is larger than ${Q}_{hmax}$, which indicates that the nonstationarity of the Hong River is larger than that of the Huai River. Therefore, more attention should be paid to flood control planning in the Hong River Basin.

In conclusion, this study provides a reasonable approach for assessing the risk of flood coincidence in the Huai River Basin under nonstationary conditions. The trends and risks of flood coincidence can be further studied to improve flood management.

## Methods

### Framework of the copula function

The copula function, which was first proposed by Sklar^8^, describes the correlation between variables. It is actually a class of function that connects joint distributions to their respective marginal distributions. Nelsen^49^ and Joe^50^ provided a theoretical introduction to copulas. Studies on practical approaches have also been developed^6,51,52^. Specifically, basic guidelines for using copulas in hydrological applications were illustrated by Favre^51^, Salvadori and De Michele^16^, and Salvadori *et al*.^53,54^ Useful and free software routines were provided and illustrated by Hofert *et al*.^55^

Environmental changes can influence both the statistical characteristics of hydrological series and the dependence structure of hydrological variables. Considering these changes, a time-varying copula should be considered in such analyses. Hence, the joint distribution of the hydrological variable pair of $({Y}_{1}^{t},{Y}_{2}^{t})$ at time $t$ can be produced as follows:

1 $$H({y}_{1}^{t},{y}_{2}^{t})=C[{F}_{1}({y}_{1}^{t}|{\theta }_{1}^{t}),{F}_{2}({y}_{2}^{t}|{\theta }_{2}^{t})|{\theta }_{c}^{t}]=C({u}^{t},{v}^{t}|{\theta }_{c}^{t})$$

where *H*(·) represents the joint cumulative distribution function (CDF) of ${Y}_{1}^{t}\,$and ${Y}_{2}^{t}$, *F*_1_ is the cumulative marginal distribution of ${Y}_{1}^{t}$, *F*_2_ is the cumulative marginal distribution of ${Y}_{2}^{t}$, C(·) is the copula function, ${{\theta }_{1}}^{t}$ and ${{\theta }_{2}}^{t}$ are the time-varying marginal distribution parameters, ${\theta }_{c}^{t}$ is the time-varying copula parameter, and the marginal probabilities ${u}^{t}\,$and ${v}^{t}\,$should obey a uniform distribution in the range of [0,1].

Under the stationary assumption, all the parameters above can set as constants:

2 $${\theta }_{1}^{t}={\theta }_{1};\,{\theta }_{2}^{t}={\theta }_{2};\,{\theta }_{c}=c$$

According to Eq. (1), the implementation of the time-varying copula consists of two steps. The first step is to analyze the univariate nonstationarity (including change point and trend tests) and select an appropriate marginal distribution. The second step is to analyze the nonstationarity of the dependence structure and select an appropriate copula function.

### Marginal distribution

#### Marginal distribution of flood occurrence dates

Flood occurrence dates can be regarded as a vector with periodic changes. The mixed von Mises distribution^56^ is a distribution commonly used to describe periodic or seasonal variables and has been proven to effectively fit flood dates^12^. The directional variable of flood occurrence dates ${x}_{i}$ can be obtained from the following relation:

3 $${x}_{i}={D}_{i}\,\frac{2\pi }{L},\,0\le {x}_{i}\le 2\pi $$

where $L$ denotes the length of the flood season and ${D}_{i}$ is the flood occurrence date. The probability density function for a mixture of *N* von Mises distribution can be produced in the following form:

4 $$f(x)=\mathop{\sum }\limits_{i=1}^{N}\,\frac{{p}_{i}}{2\pi {I}_{0}({\kappa }_{i})}{\exp }^{[{\kappa }_{i}\cos (x-{u}_{i})]}$$

where ${u}_{i}$ and ${\kappa }_{i}$ are location and scale parameters, respectively; ${p}_{i}$ is the weight of the probability density; $N=2$ in this study; and ${I}_{0}$ is the Bessel function. In this study, parameters were estimated by the maximum likelihood estimation method, and the approximate Monte Carlo goodness-of-fit test *p*-values (based on Kolmogorov–Smirnov statistics) were calculated to assess the validity of the assumption that the flood occurrence dates followed the selected distribution.

#### Marginal distribution of flood magnitudes

In this study, five widely used distributions, including the gamma (GA), Weibull (WEI), lognormal (LOGNO), Gumbel (GU), and generalized extreme value (GEV) distributions, were selected to describe the flood magnitudes (Table S1). Then, based on the GAMLSS model proposed by Rigby and Stasinopoulos^36^, the time-varying marginal distribution was determined; such distributions are widely used in frequency analyses of nonstationary hydrological series.

Taking a three-parameter model with one explanatory variable as an example, if the response variable ${y}^{t}$ follows the distribution function ${F}_{y}=F({y}^{t}|{\mu }_{t},{\sigma }_{t},{\nu }_{t})$ at time $t\,(t=1,2,\cdot \cdot \cdot \cdot \cdot \cdot n)$, then each parameter can be described as a linear function of the explanatory variable ${x}^{t}$ via a monotonic link function *g*_*k*_(·) as follows:

5 $${g}_{1}({\mu }_{t})={a}_{1}+{b}_{1}{x}^{t}$$

6 $${g}_{2}({\sigma }_{t})={a}_{2}+{b}_{2}{x}^{t}$$

7 $${g}_{3}({\nu }_{t})={a}_{3}+{b}_{3}{x}^{t}$$

where ${a}_{k}$ and ${b}_{k}(k=1,2,3)$ are the parameters of GAMLSS.

In practice, only the location parameter ${\mu }_{t}$ and scale parameter ${\sigma }_{t}$ are considered to be associated with the explanatory covariate. In other words, either one of these variables is time varying or both are time varying. The shape parameter ${\nu }_{t}$ is treated as a constant (i.e., ${b}_{3}=0$). The stationary distribution can be obtained by assuming that the parameters are independent of the explanatory variable (i.e., ${b}_{1}={b}_{2}={b}_{3}=0$).

The optimal distribution can be selected from the candidates by comparing the value of the Akaike information criterion (AIC). All computational processes were implemented in the R package “gamlss”.

### Copula with time-varying parameters

In multivariate hydrological frequency analysis, the Archimedean copula is widely used due to its flexibility in structural form^49^. Here, three simple single-parameter copulas, including the Gumbel copula (GH), Clayton copula (CL), and Frank copula (FR), were used as candidates to model the time-varying dependence structure (Table S4).

The time-varying copula function assumes that the copula parameters obey a time-varying process and have time-varying characteristics. In this study, a process similar to ARMA (1, *q*)^35^ was used to describe the time-varying nature of the parameters:

8 $${\tau }_{c}^{t}=\varLambda (\omega +\beta {\tau }_{t-1}+\alpha \frac{1}{q}\,\mathop{\sum }\limits_{j=1}^{q}({u}_{t-j}-{v}_{t-j}))$$

where $\varLambda ()\,$is a transformation function that considers the correlation with ${\rho }_{t}$ in the definition interval (0, 1) in this study, $\varLambda (x)=\frac{1}{1+exp(-x)}$, and ${\tau }_{c}^{t}$^57^ can be converted to ${\theta }_{c}^{t}$^58^.

In this paper, the ARMA model with a lag of 10 orders was adopted (*q =* 10). Two-step pseudo maximum likelihood estimation was adopted for parameter estimation. First, the marginal distribution parameters were calculated, and the empirical distribution was then used instead of the marginal distribution to determine the logarithmic likelihood function of the following equation. A two-step method was used to estimate the maximum likelihood.

9 $$L({\theta }_{c}^{t})={\sum }_{t}^{T}lnc({\widehat{F}}_{X}(x),{\widehat{F}}_{Y}(y);{\theta }_{c}^{t})L({\theta }_{c}^{t})={\sum }_{t}^{T}lnc({\widehat{F}}_{X}(x),{\widehat{F}}_{Y}(y);{\theta }_{c}^{t})$$

where c(·) indicates the density of the copula function and ${\widehat{F}}_{X}(x)$ and ${\widehat{F}}_{Y}(y)$ represent the empirical marginal distributions. The parameters can be obtained by combining Eqs. (1) and (9). Because the test method of calculating the “distance” between the fitted copula and the empirical copula is not suitable for time-varying copulas^59^, the Rosenblatt probability integral transformation was used to test the goodness-of-fit of the time-varying copulas^60^. The optimum copula was selected according to the minimum AIC.

### Analysis of flood coincidence risk

The term coincidence refers to the simultaneous occurrence of floods in two (or more) rivers, which can be measured by the exceedance probabilities of flood events. As flood events are characterized by flood occurrence dates and magnitudes, both of these factors should be considered.

First, the flood occurrence dates were defined as the occurrence dates of the AMDF in the Huai River or Hong River. Then, the daily coincidence probabilities for the flood occurrence dates were described by the following mathematical equation:

10 $${P}_{i}=P({t}_{i}\le {T}_{h}\le {t}_{i+1},{t}_{i}+{\tau }_{hb}-\varDelta t\le {T}_{b}\le {t}_{i}+{\tau }_{hb}+\varDelta t)$$

11 $${P}_{T}={\sum }_{i=1}^{n}{P}_{i}$$

where $i$ indicates the ${i}^{th}$ day of the flood season and $\varDelta t$ indicates the time interval between the response in the two rivers. Because the maximum daily flow in the two rivers rarely occurs on the same day, we defined $\varDelta t=1$ as a flood coincidence event in this study, and ${\tau }_{hb}$ indicates the difference in propagation time between the two stations (the difference in propagation time between the Huaibin and Bantai stations is 16 hours). Additionally, *n* indicates the length of the flood season, and ${P}_{T}$ is the annual coincidence probability of flood occurrence dates.

Second, the series of AMDFs was selected to fit the joint distribution of flood magnitudes, assuming that the random variables of AMDF in Huaibin ${Q}_{h}$ obey the ${F}_{H}$ distribution and the random variables of AMDF in Bantai ${Q}_{h}$ obey the ${F}_{B}$ distribution. Then, the exceedance probability of flood coincidence for a given flood magnitude in the ${t}^{th}$ year can be defined as:

12 $${P}_{Q}^{t}={P}^{t}({Q}_{h}\ge {q}_{h},{Q}_{b}\ge {q}_{b})=1-{F}_{H}({q}_{h}|{\theta }_{1}^{t})-{F}_{B}({q}_{b}|{\theta }_{2}^{t})+C({F}_{H}({q}_{h}|{\theta }_{1}^{t}),{F}_{B}({q}_{b}|{\theta }_{2}^{t})|{\theta }_{c}^{t})$$

where ${q}_{h}$ and ${q}_{b}$ are the designed flows, C(·) represents the copula function with time-varying parameters, ${P}_{Q}^{t}$ is the exceedance probabilities for coincident flood magnitudes, and ${Q}_{h}$ and ${Q}_{b}$ are the flood magnitudes, namely, the AMDFs sampled by the AM method.

Then, based on the hypothesis that the flood occurrence dates and flood magnitudes are independent of each other (i.e., the Kendall coefficient is less than 0.05), the annual flood coincidence probabilities ${P}_{t}$ for given flood magnitudes in the ${t}^{th}$ year can be stated as AND-joint exceedance probabilities:

13 $${P}_{t}={\sum }_{i=1}^{n}{P}_{i}{P}_{Q}^{t}={P}_{T}{P}^{t}({Q}_{h}\ge {q}_{h},{Q}_{b}\ge {q}_{b})$$

This equation describes the probability that ${Q}_{h}$ is greater than ${q}_{h}$ and ${Q}_{b}$ is greater than ${q}_{b}$ when the occurrence dates of these two flows differ by one day.

Finally, the impacts of flood coincidence on the lower reaches of the basin were analyzed using the “most likely” design^38^. When ${P}_{t}$ is fixed as a constant in the range of (0,1), there are many possible combinations of (${Q}_{h}$, ${Q}_{b}$), where ${P}_{Q}^{t}$ is also a constant because of the constant nature of ${P}_{T}$. Among these combinations, the “most likely” design reflects the combination with the highest probability, as defined in Eqs. (14–15).

14 $$({\mu }_{m}^{t},{\nu }_{m}^{t})=argmax\,{f}_{c}({F}_{H}^{-1}(\mu |{\theta }_{1}^{t}),{F}_{B}^{-1}(\nu |{\theta }_{2}^{t})|{\theta }_{c}^{t})$$

where ${F}_{H}^{-1}$(·) and ${F}_{B}^{-1}$(·) are the inverse functions of the marginal distributions of ${Q}_{h}$ and ${Q}_{b}$ with time-varying parameters, respectively; $\,{f}_{c}$ is the joint probability density with time-varying parameter ${\theta }_{c}^{t}$; and $\mu$ and $\nu$ are independent of each other and subject to a uniform distribution in the range of [0, 1]. Then, the “most likely” design in the ${t}^{th}$ year can be calculated by using the inverse of the CDFs of the marginal distributions:

15 $${Q}_{hmax}^{t}={F}_{H}^{-1}({\mu }_{m}^{t}|{{\boldsymbol{\theta }}}_{1}^{t})\,and\,{Q}_{bmax}^{t}={F}_{B}^{-1}({v}_{m}^{t}|{{\boldsymbol{\theta }}}_{2}^{t})$$

## Supplementary information

Supplementary Information.
