# Supplementary material for: Nonstationary flood coincidence risk analysis using time-varying copula functions
Source: Sci Rep. 2020 Feb 25;10:3395. doi: 10.1038/s41598-020-60264-3 (PMC7042327; doi:10.1038/s41598-020-60264-3)
Supplement: Supplementary file 1 — Supplementary Information. [file 41598_2020_60264_MOESM1_ESM.docx]

**Nonstationary flood coincidence risk analysis using time-varying copula functions**

**Ying Feng ^1^, Peng Shi ^1, 2^, Simin Qu ^1^*, Shiyu Mou^1^, Chen Chen^1^and Fengcheng Dong^1^**

^1^ College of Hydrology and Water Resources, Hohai University, Nanjing 210098, China;

[fengying@hhu.edu.cn](mailto:fengying@hhu.edu.cn) (Y.F); ship@hhu.edu.cn (P.S); [wanily@hhu.edu.cn (S.Q)](mailto:wanily@hhu.edu.cn%20(S.Q)); ariesmsy@hhu.edu.cn (S.M);

Johnnychenhhu@gmail.com (C.C);dfcheng93@126.com (F.D)

^2^ State Key Laboratory of Hydrology-Water Resources and Hydraulic Engineering,

Hohai University, Nanjing 210098, China

***** Correspondence: [wanily@hhu.edu.cn](mailto:wanily@hhu.edu.cn%20(S.Q)) ; Tel.: +86-138-5171-3291

**Supplementary Table S1.** Probability density functions of the marginal distributions and their link functions.

| **Distribution** | **Probability density function** | **Link function** | |
| --- | --- | --- | --- |
|  |  | $g\left( u \right)$ | $g\left( \sigma\right)$ |
| Gamma | $f(x)=\frac{1}{(\sigma^{2}\mu)^{1/\sigma^{2}}}\frac{x^{(1/\sigma^{2})-1}e^{-x/(\sigma^{2}\mu)}}{\Gamma(1/\sigma^{2})}$ | $\ln\left( u \right)$ | $\ln\left( \sigma\right)$ |
| Weibull | $f(x)=\frac{\sigma x^{\sigma-1}}{\mu^{\sigma}}\exp\left[ -(\frac{x}{\mu})^{\sigma} \right]$ | $\ln\left( u \right)$ | $\ln\left( \sigma\right)$ |
| Lognormal | $f(x)=\frac{1}{\sqrt{2\pi\sigma^{2}}}\frac{1}{x}\exp\left\{ -\frac{\left[ \log(x)-\mu\right]^{2}}{2\sigma^{2}} \right\}$ | $\ln(u)$ | $\ln\left( \sigma\right)$ |
| Gumbel | $f(x)=\frac{1}{\sigma}\exp\left[ (\frac{x-\mu}{\sigma})-\exp(\frac{x-\mu}{\sigma}) \right]$ | $u$ | $\ln\left( \sigma\right)$ |
| GEV | $f(x)=\frac{1}{\sigma}(1+\frac{\nu(x-u)}{\sigma})^{-1/(\nu-1)}\exp\left\{ -\left[ 1+\frac{\nu(x-u)}{\sigma} \right]^{-1/\nu} \right\}$ | $u$ | $\ln\left( \sigma\right)$ |

**Supplementary Table S2.**  Performance of the five distributions in fitting the two series under stationary assumption

| **Series** | **Function** | **parameters** | | | ***p*-Value** | **AIC** |
| --- | --- | --- | --- | --- | --- | --- |
|  |  | $\mu$ (s.e.) | $\sigma$ (s.e.) | $\nu$ (s.e.) |  |  |
| *Q_h_* | GA | 1856(182.3) | 1.455(0.112) |  | 0.73 | 1009.9 |
|  | GU | 1839(148.4) | 1375(190.4) |  | 0.17 | 1099.9 |
|  | WEI | 2961(307.3) | 1.349(0.129) |  | 0.51 | 1010.6 |
|  | LOGNO | 2079(207.2) | 2.061(0.169) |  | 0.92 | 1012.7 |
|  | GEV | 1696(189.8) | 1244(151.0) | 0.194(0.116) | 0.50 | 1010.7 |
| *Q_b_* | GA | 494.7(29.25) | 1.874(0.179) |  | 0.70 | 885.0 |
|  | GU | 631.2(67.42) | 483.7(51.97) |  | 0.32 | 916.6 |
|  | WEI | 1018(95.53) | 1.486(0.157) |  | 0.57 | 884.1 |
|  | LOGNO | 686.3(72.80) | 2.332(0.178) |  | 0.98 | 891.3 |
|  | GEV | 609.4(76.22) | 465.3(60.14) | 0.085(0.159) | 0.43 | 892.5 |

**Supplementary Table S3.** Performance of the five distributions in fitting the flow series under nonstationary assumption

| **Probability**  **distribution** | | **Distribution parameters** | | | **AIC** | **SBC** | ***p*-Value** |
| --- | --- | --- | --- | --- | --- | --- | --- |
|  |  | $\mu$ | $\theta$ | $\nu$ |  |  |  |
| *Q_h_* | GA | $\exp(8.068)$ | $\exp(0.166-0.114P_{h})$ |  | 950.3 | 958.4 | 0.59 |
|  | GU | $1637$ | $\exp(5.008-0.576P_{h})$ |  | 1029 | 1036 | 0.12 |
|  | WEI | $\exp(7.894)$ | $\exp(0.607-0.065P_{h})$ |  | 946.1 | 954.3 | 0.89 |
|  | LOGNO | $\exp(7.867)$ | $\exp(0.441-0.154P_{h})$ |  | 1009 | 1015 | 0.64 |
|  | GEV | $1703$ | $\exp(6.790-0.096P_{b})$ Exp(6.79+964.7*P)* | 0.057 | 1011 | 1019 | 0.68 |
| *Q_b_* | GA | $\exp(5.440+0.308P_{b})$ | $\exp(-0.024-0.154P_{b})$ |  | 844.6 | 852.8 | 0.32 |
|  | GU | $\exp(-392.0+371.2P_{b})$ | $\exp(5.607+0.128P_{b})$ |  | 872.7 | 881.0 | 0.24 |
|  | WEI | $\exp(5.554+0.309P_{b})$ | $\exp(0.040+0.166P_{b})$ |  | 846.6 | 854.8 | 0.41 |
|  | LOGNO | $\exp(5.131+0.346P_{b})$ | $\exp(0.036-0.153P_{b})$ |  | 846.6 | 854.7 | 0.62 |
|  | GEV | $417.4+70.18P_{b}$ | $\exp(5.448+0.170P_{b})$ | -0.205 | 879.9 | 890.2 | 0.57 |

**Supplementary Table S4.** The description of the Archimedean copulas.

| **Copula** | $\boldsymbol{C(u,v)}$ | $\boldsymbol{\theta}$ |
| --- | --- | --- |
| Gumbel | $\exp\left\{ -\left[ (-\ln u)^{\theta}+(-\ln v)^{\theta} \right]^{1/\theta} \right\}$ | $\theta\geq1$ |
| Clayton | $\max(\left[ u^{-\theta}+v^{-\theta}-1 \right]^{-\frac{1}{\theta}},0)$ | $\theta>0$ |
| Frank | $-\frac{1}{\theta}\ln\left[ 1+\frac{(e^{-\theta u}-1)(e^{-\theta v}-1)}{e^{-\theta}-1} \right]$ | $\theta\neq0$ |
